# Comparative Congenital Cardiac Catheterization Registry Analysis From the United States and Low- and Middle-Income Countries

**DOI:** 10.1016/j.jacadv.2025.101649

**Published:** 2025-03-20

**Authors:** Fatima Ali, Mary J. Yeh, Fiona E. Walshe, Lisa Bergersen, Kimberlee Gauvreau, Oliver M. Barry, Brian A. Boe, Ralf J. Holzer, Rik De Decker, Kathy Jenkins, Jacqueline Kreutzer, Raman Krishna Kumar, John Lozier, Michael L. O’Byrne, Igor Polivenok, Miguel Ronderos, Babar Hasan, Brian P. Quinn

**Affiliations:** a Division of Cardiothoracic Sciences, Sindh Institute of Urology and Transplantation (SIUT), Karachi, Pakistan; b Department of Cardiology, Boston Children's Hospital, Boston, Massachusetts, USA; c Division of Pediatric Cardiology, New York-Presbyterian-Morgan Stanley Children's Hospital, Columbia University Medical Center, New York, New York, USA; d Heart Institute, Joe DiMaggio Children's Hospital, Hollywood, Florida, USA; e Pediatric Heart Center, University of California Davis Medical Center Children's Hospital, Sacramento, California, USA; f Division of Pediatric Cardiology, Department of Pediatrics and Child Health, Red Cross War Memorial Children's Hospital, Cape Town, South Africa; g Division of Cardiology, Department of Pediatrics, University of Pittsburgh School of Medicine, UPMC Children's Hospital of Pittsburgh, Pittsburgh, Pennsylvania, USA; h Division of Cardiology, Department of Pediatrics, Amrita Institute of Medical Sciences and Research Centre, Kochi, India; i Division of Cardiology, Department of Pediatrics, UH Rainbow Babies and Children's Hospital, Cleveland, Ohio, USA; j Division of Cardiology, The Children's Hospital of Philadelphia and Department of Pediatrics Perelman School of Medicine, Philadelphia, Pennsylvania, USA; k Zaitcev V.T. Institute of General and Urgent Surgery and William Novick Global Cardiac Alliance, Kharkiv, Ukraine; l Fundacion Cardioinfantil de Bogota, Bogota, Colombia

**Keywords:** congenital cardiac catheterization, comparative effectiveness, congenital heart disease, patient-centered outcomes research, pediatric cardiology

## Abstract

**Background:**

Disparities in congenital heart disease care exist between high-income and low- and middle-income countries (LMICs), likely extending to congenital cardiac catheterization (CCC).

**Objectives:**

This study compares patient characteristics and outcomes of CCC in the U.S.-based Congenital Cardiac Catheterization Project on Outcomes (C3PO) and the International Quality Improvement Collaborative—Congenital Heart Disease Catheterization Registry (IQIC-CHDCR) from LMICs.

**Methods:**

The analysis included all CCC procedures recorded in C3PO (19 sites) and IQIC-CHDCR (19 sites) from 2019 to 2022. Patient and procedural characteristics, resource utilization, and outcomes were compared.

**Results:**

A total of 28,957 C3PO and 6,485 IQIC-CHDCR cases were analyzed. Single ventricle patients accounted for 30% of C3PO and 13% of International Quality Improvement Collaborative (IQIC), with high-risk procedures (procedural risk in congenital cardiac catheterization 3-5) performed more frequently in C3PO (42% vs 23%). Median procedure duration was longer in C3PO (1.5 vs 0.8 hours). Clinically meaningful adverse event (CMAE) rates were higher in C3PO (3.9% vs 1.5%), though mortality was comparable (0.5% vs 0.7%). Risk-adjusted analysis showed a lower ratio in IQIC for both CMAE (0.50; 95% CI: 0.39-0.62) and severity level 4/5 events (0.71; 95% CI: 0.52-0.96). However, failure-to-rescue rates were higher in IQIC (7.1% vs 2.1%).

**Conclusions:**

The harmonized databases facilitated direct comparison of CCC practices, revealing more complex patients and resource-intensive procedures in C3PO, while the IQIC cohort demonstrated lower CMAE rates but a slightly higher mortality rate. These findings emphasize the need for further risk adjustment modeling for LMICs and identify areas to enhance global resource access and patient outcomes.

Congenital heart disease (CHD) affects millions globally, with 90% of cases occurring in low- and middle-income countries (LMICs), where mortality rates from CHD are significantly higher than in high-income countries (HICs) due to late diagnosis, limited access to specialized care, and inadequate health care infrastructure.^1^, ^2^, ^3^ Congenital cardiac catheterization (CCC) is a critical diagnostic and therapeutic tool for CHD, yet significant differences in postcatheterization outcomes between HICs and LMICs persist, presumably due to late diagnosis, limited access to care, and the lack of specialized cardiac centers and expertise.^4^, ^5^, ^6^ It is critical to better understand these differences to best identify modifiable areas to help guide efforts at identifying actionable items.

While recent studies have demonstrated the safety and efficacy of cardiac catheterization in LMICs,^7^ there remains a gap in comparative data on patient populations, case mix, and outcomes between LMICs and HICs. A lack of comprehensive and representative data from diverse LMIC programs has hindered our understanding of these disparities, limiting opportunities to improve care. This study seeks to address this gap by providing a detailed comparison of patient and procedural characteristics, resource utilization, and outcomes between LMIC and HIC settings.

This study utilizes data from 2 large registries: the Congenital Cardiac Catheterization Project on Outcomes (C3PO) from the United States and the International Quality Improvement Collaborative—Congenital Heart Disease Catheterization Registry (IQIC-CHDCR) from LMICs, which was developed to mirror the success of the C3PO registry.^8^

Leveraging the harmonized data entry platform and similar data elements of these registries, we aim to conduct a comprehensive analysis of case mix, procedural characteristics, and outcomes to enhance understanding of differences in CCC practices between HICs and LMICs. By understanding these differences, we can begin to identify gaps and opportunities for improving care and access to catheter-based interventions globally.

## Methods

### Study design and population

This is a descriptive, retrospective comparative report utilizing data from 2 registries, IQIC-CHDCR and C3PO. IQIC-CHDCR collects data from LMICs, while C3PO is a U.S.-based registry and does not collect data from other HICs or LMICs. The study encompasses all pediatric and/or CCCs, including diagnostic and interventional cases, performed between January 1, 2019, and December 31, 2022, recorded in either registry. Participating sites are listed in Supplemental Table 1. Approval for the study was obtained from the Institutional Review Board at Boston Children's Hospital, USA, the sponsor site for both registries. Data collection and analysis on the same web-based platform ensures deidentified data for security and privacy. International Quality Improvement Collaborative (IQIC) is free of cost for its participating sites, while C3PO charges annually.

All participating sites in both registries underwent virtual audits annually to ensure data accuracy and verify complete case entry, as detailed previously.^9^ In the C3PO registry, an annual audit was conducted on up to 10% of a site's total case volume, with a maximum of 20 cases. Similarly, the IQIC-CHDCR audited 10% of a site's total case volume, examining key predictor risk variables and outcomes, including the occurrence and severity of adverse events (AEs) (Supplemental Table 2). The analysis only includes sites that successfully passed their respective data audits.

### Data elements

#### Patient characteristics

Patient characteristics included age, weight at the time of catheterization (in kilograms), sex, the presence of any known or suspected genetic syndrome, single ventricle (SV) functional physiology, and noncardiac comorbidities (including chronic lung disease, renal insufficiency, coagulation disorder, or other). Any cardiac procedures (catheterization and/or surgery) within 90 days before the procedure were recorded as dichotomous variables. Preprocedure cardiac status (PCS) was categorized as 1, 2, or 3 as determined by clinical assessment and noninvasive diagnostic methods such as echocardiography.^10^

#### Characteristics of clinical care

Clinical care information includes admission data such as whether the procedure was electively scheduled or involved an unplanned hospitalization and if the patient was admitted more than 48 hours before the procedure. Nursing assignments (nurse-to-patient ratio as 1:≤2, 1:3, 1:4) for admitted patients were documented at 24 and 48 hours postprocedure. Discharge data included the initial postcath location (outpatient after 6 hours, general floor, step-down unit, intensive care unit [ICU], or death in the lab), any unplanned hospitalization, and whether the patient was discharged more than 48 hours after the procedure.

#### Procedural characteristics

Cases were broadly classified as either diagnostic or interventional, and interventional cases were recorded as procedures involving isolated or multiple interventions. Cases were categorized based on the procedural risk in congenital cardiac catheterization (PREDIC^3^T) case type risk category (as 1, 2, 3, 4, 5, or "unassigned"), a classification previously developed using C3PO data.^9^ Procedure duration was calculated as the time from sheath-in to sheath-out (in minutes), excluding the time dedicated to managing any AEs. This time was retrieved from the time to manage AE variable entered by participating sites and from the description of AE. Case durations below the 1st percentile or above the 99th percentile were excluded. Radiation exposure surrogate data, measured as dose area product, were collected. For this study, we measured the frequency of dose area product entry as either "recorded" or "not recorded" by the participating sites to account for missing data.

#### Procedural resource utilization

Data regarding the supportive resources used during the procedure were recorded. These included the level of sedation employed (general anesthesia). This time was retrieved from the time to manage AE variable entered by participating sites and from the description of AEs, intravenous (IV) sedation, or local/oral/no sedation, airway management (tracheostomy or endotracheal tube, other airway support [no endotracheal tube], or natural airway), ventilation practices (room air only, supplemental oxygen, and/or any nitric oxide), continuous IV medications (supportive or essential vasoactive, antiarrhythmics, prostaglandin E, and/or any other continuous medications), the presence of any cardiac mechanical support (including extracorporeal membrane oxygenation [ECMO] and/or other supports), as well as the presence of drains and lines (central venous or arterial lines). Additionally, the administration of a blood transfusion either during or within 72 hours of the procedure was captured.

#### Hemodynamic variables and hemodynamic vulnerability score

Hemodynamic variables collected during the procedure were recorded, including systemic arterial saturation, mixed venous saturation, pulmonary artery (PA) pressure (as systolic PA pressure for biventricular patients and mean PA pressure for SV patients), systemic ventricle end-diastolic pressure, ratio of pulmonary to systemic flow (Qp:Qs), and pulmonary vascular resistance. Values were captured as either normal or abnormal, based on established thresholds. Hemodynamic vulnerability score (HVS), as 0, 1, 2, or ≥3, was recorded.^9^

#### Procedure outcomes

Any AEs occurring during or after the procedure, recorded and described in the database, were analyzed. Individual AEs were categorized by type (eg, arrhythmia, sedation/airway problem). Life-threatening events not resulting in patient death were classified as severity level 4, following the International Pediatric and Congenital Cardiac Code definitions.^11^ Level 3 events were further categorized into three tiers (3a, 3b, or 3c) based on clinical impact, using previously established definitions.^12^^,^^13^ Clinically meaningful adverse events (CMAEs) encompassed AEs of severity levels 3b, 3c, 4, and 5. Under the supervision and guidance of interventional pediatric cardiologists (L.B. and B.P.Q.), the data management personnel reviewed AE severity classification to ensure consistent severity scoring across all centers.

In this report, we present the highest-level AE in a case and the AE rates within PREDIC^3^T categories and case types.

Death within 72 hours of the catheterization procedure for any reason was analyzed, and each case was individually reviewed by data management personnel (alongside L.B. and B.P.Q.) to assess any potential attributability of an AE to the death. Additionally, an analysis was conducted to examine the relationship between AEs and whether the lack of availability of backup/rescue support in LMICs (ie, ECMO) had an effect on the outcome.

### Statistical analysis

Patient and procedural characteristics were summarized using frequencies and percentages for categorical variables, while continuous variables were described using median and IQR (25th and 75th percentile). To assess differences between the 2 cohorts in case mix and outcomes, the chi-square test was employed for categorical data, and the Wilcoxon rank-sum test for continuous variables. Given the large sample size and many comparisons performed, statistical significance was set at *P* < 0.001. In addition to being summarized as a continuous variable, age was categorized into 4 distinct groups: ≤30 days, 31 days to <1 year, 1 to 18 years, and ≥19 years. Procedure duration was also classified into 4 categories: <1 hour, 1 to 2 hours, 2 to 3 hours, and ≥3 hours. The HVS was computed by aggregating abnormal threshold values (+1 or +2) of hemodynamic parameters obtained during catheterization.^9^

In addition to comparing the percentages of patients experiencing any AE between the 2 cohorts, we also counted the total numbers of events and calculated AE rates per 1,000 patients. Furthermore, “failure to rescue” was defined as the number of deaths as a result of AE divided by the number of patients with a CMAE (severity level 3bc/4/5) event. The CHARM II risk adjustment method was used to compare AE rates accounting for case mix differences in PREDIC^3^T case type risk category, HVS, and age; C3PO was used as the benchmark cohort, and a standardized adverse event ratio (SAER) was calculated for IQIC-CHDCR.^12^

## Results

The analysis encompassed 28,957 cases from 19 C3PO sites and 6,485 cases from 19 IQIC-CHDCR sites (Central Illustration).

**Central Illustration:**
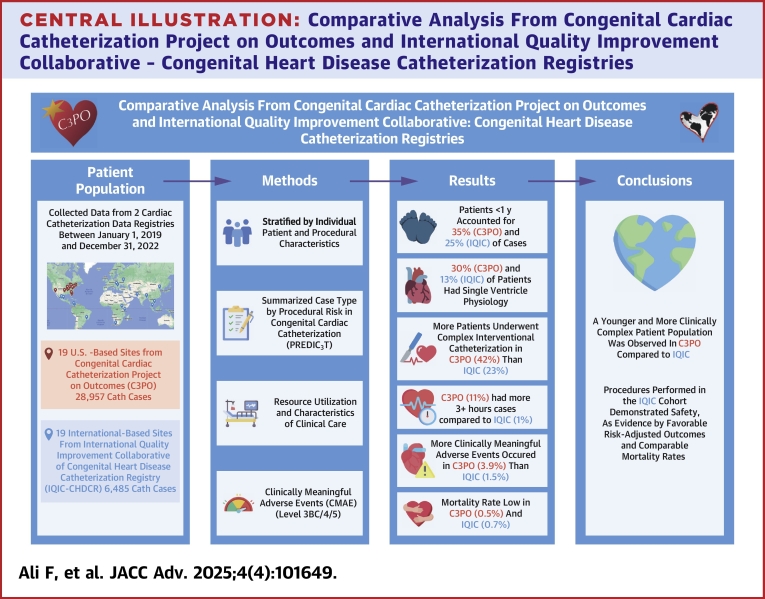
**Comparative Analysis From Congenital Cardiac Catheterization Project on Outcomes and International Quality Improvement Collaborative – Congenital Heart Disease Catheterization Registries** C3PO = Congenital Cardiac Catheterization Project on Outcomes; IQIC-CHDCR = International Quality Improvement Collaborative—Congenital Heart Disease Catheterization Registry; PREDIC_3_T = procedural risk in congenital cardiac catheterization.

### Comparison of patient characteristics between U.S. centers and LMIC

Significant differences in patient characteristics were observed between the 2 cohorts. Notable variations included a higher percentage of individuals in the younger age group (<1 year) in C3PO (35%) compared to IQIC (35% vs 25%). Additionally, procedures performed in babies weighing <3 kg were more frequent in C3PO than IQIC (7% C3PO, 3% IQIC). Patients with underlying genetic syndromes (15% C3PO, 6% IQIC) and those with noncardiac problems (23% C3PO, 4% IQIC) were also reported more frequently in C3PO than in IQIC. Notably, nearly one-third of the C3PO cohort consisted of patients with a SV (30%), more than double the proportion in IQIC (13%). A significantly higher proportion of the C3PO cohort had recent cardiac procedures (18% in C3PO and 5% in IQIC). Furthermore, more patients were in the lower risk group of PCS 1 in IQIC (60%) compared to C3PO (46%), in contrast to the mid-level risk group of PCS 2, which consisted of 24% of IQIC and 35% of C3PO patients. The highest risk group (PCS 3) did not show any significant differences, with 19% in C3PO and 16% in IQIC (Table 1).

**Table 1:** Patient Characteristics

	C3PO (n = 28,957)	IQIC-CHDCR (n = 6,485)	*P* Value
Age (y)	3 (0.3, 12)	3 (1, 9)	
≤30 d	2,955 (10%)	367 (6%)	<0.001
31 d to < 1 y	7,339 (25%)	1,242 (19%)	
1 to 18 y	15,203 (53%)	4,271 (66%)	
≥19 y	3,460 (12%)	605 (9%)	
Weight (kg) (n = 28,956, n = 6,484)	14 (6, 42)	14 (8, 28)	
Weight <3 kg	1,914 (7%)	181 (3%)	<0.001
Sex male (n = 28,956, n = 6,484)	15,561 (54%)	3,058 (47%)	<0.001
Genetic syndrome	4,481 (15%)	415 (6%)	<0.001
Single ventricle (n = 28,905, n = 6,484)	8,629 (30%)	874 (13%)	<0.001
Any noncardiac problem	6,559 (23%)	286 (4%)	
Coagulation disorder	122 (<1%)	4 (<1%)	<0.001
Chronic lung disease	1,681 (6%)	36 (1%)	
Renal insufficiency	322 (1%)	8 (<1%)	
Other	4,434 (15%)	238 (4%)	
Cardiac procedure within 90 d	5,205 (18%)	330 (5%)	
Cardiac catheterization	3,446 (12%)	210 (3%)	<0.001
Cardiac surgery	3,535 (12%)	192 (3%)	
Preprocedure cardiac status (n = 25,311, n = 6,469)			
PCS 1	11,744 (46%)	3,886 (60%)	<0.001
PCS 2	8,866 (35%)	1,581 (24%)	
PCS 3	4,701 (19%)	1,002 (16%)	

Values are median (IQR) or n (%).

C3PO = Congenital Cardiac Catheterization Project on Outcomes; IQIC-CHDCR = International Quality Improvement Collaborative—Congenital Heart Disease Catheterization Registry; PCS = preprocedure cardiac status.

A greater number of cases were electively scheduled in IQIC than in C3PO, 94% vs 79% in IQIC and C3PO, respectively. At 48 hours postprocedure, approximately half the proportion of IQIC patients had a nursing assignment of 1:≤2 compared to C3PO (13% and 25%, respectively).

Patients admitted >48 hours preprocedure accounted for 27% of the C3PO cohort and 11% of the IQIC cohort. Similarly, patients discharged >48 hours postprocedure were more prevalent in C3PO (34% C3PO, 21% IQIC).

Additionally, a slightly increased frequency of patients who went to the ICU postprocedure (36% C3PO, 32% IQIC) and stepdown unit (13% C3PO, 10% IQIC) was noted in C3PO. However, deaths occurring in the cath lab showed similar results, accounting for <1% in both cohorts (Table 2).

**Table 2:** Characteristics of Clinical Care

	C3PO (n = 28,957)	IQIC-CHDCR (n = 6,485)	*P* Value
Admission Information			
Elective	22,951 (79%)	6,119 (94%)	<0.001
Admit >48 h prior to Cath	7,892 (27%)	742 (11%)	<0.001
Nursing assignment (nurse:patient ratio) 24 h postprocedure			
1:≤2	8,731 (30%)	2,464 (38%)	<0.001
1:3	3,515 (12%)	1,115 (17%)	
1:4	6,461 (22%)	2,621 (40%)	
Discharged/home	10,250 (35%)	285 (4%)	
Nursing assignment (nurse:patient ratio) 48 h postprocedure (2021-2022 only)			
1:≤2	4,020 (25%)	405 (13%)	<0.001
1:3	526 (3%)	397 (12%)	
1:4	728 (5%)	496 (15%)	
Discharged/home	10,938 (67%)	1,907 (60%)	
Discharge information			
Discharge >48 h postcath	9,831 (34%)	1,357 (21%)	<0.001
Unplanned hospitalization postcath (n = 28,955, 6,485)	818 (3%)	342 (5%)	<0.001
Postcath location (n = 28,940, n = 6,484)			
Intensive care unit	10,383 (36%)	2,044 (32%)	<0.001
Step down unit	3,663 (13%)	643 (10%)	
General floor	7,607 (26%)	3,723 (57%)	
Outpatient	7,281 (25%)	72 (1%)	
Death in lab	6 (<1%)	2 (<1%)	

Values are n (%).

Abbreviations as in Table 1.

### Procedural characteristics

Diagnostic procedures accounted for 37% of all cases in IQIC and 33% in C3PO. The low-risk PREDIC^3^T 1 to 2 categories cases (72% IQIC, 50% C3PO), such as atrial septal defect/patent foramen ovale device closures (12.3% IQIC, 5.2% C3PO) and patent ductus arteriosus (PDA) device closures (20.8% IQIC, 10.5% C3PO), were performed more frequently in IQIC (Supplemental Table 3). Complex cases involving multiple interventions were dominant in the C3PO cohort (12% C3PO, 2% IQIC) (Table 3).

**Table 3:** Procedure Characteristics

	C3PO (n = 28,957)	IQIC-CHDCR (n = 6,485)	*P* Value
Procedure type			
Diagnostic	9,541 (33%)	2,404 (37%)	
Interventional, isolated procedure	16,012 (55%)	3,935 (61%)	<0.001
Interventional, complex procedure	3,404 (12%)	142 (2%)	
PREDIC^3^T case type risk category			
Category 1	7,217 (25%)	2,208 (34%)	<0.001
Category 2	7,252 (25%)	2,477 (38%)	
Category 3	4,987 (17%)	670 (10%)	
Category 4	4,383 (15%)	415 (6%)	
Category 5	2,652 (9%)	372 (6%)	
Not assigned	2,466 (9%)	343 (5%)	
Case duration (n = 28,203, n = 6,335)∗			
Case duration (h)	1.5 (0.9, 2.2)	0.8 (0.6, 1.2)	
<1 h	7,661 (27%)	3,620 (57%)	<0.001
1-2 h	11,555 (41%)	2,343 (37%)	
2-3 h	5,799 (21%)	329 (5%)	
≥3 h	3,188 (11%)	43 (1%)	
Radiation			
Data recorded	27,770 (96%)	4,893 (75%)	<0.001
Procedural resource utilization			
Sedation			
General anesthesia	26,884 (93%)	4,643 (72%)	<0.001
IV sedation	1,883 (6%)	1,644 (25%)	
None or local/oral	190 (1%)	198 (3%)	
Airway management (n = 28,952, n = 6,485)			
ETT/tracheostomy	26,649 (92%)	4,539 (70%)	<0.001
Airway support (no ETT)	325 (1%)	491 (8%)	
Natural airway	1,978 (7%)	1,455 (22%)	
Ventilation (n = 28,922, n = 6,465)			
Room air only	16,083 (56%)	3,106 (48%)	<0.001
Supplemental oxygen	11,448 (40%)	3,317 (51%)	
Any nitric oxide	1,391 (5%)	42 (1%)	
IV medications			
Any IV medication	7,140 (25%)	418 (6%)	<0.001
Vasoactive - supportive	4,558 (16%)	221 (3%)	
Vasoactive - essential	1,684 (6%)	55 (1%)	
Antiarrhythmics	135 (<1%)	9 (<1%)	
PGE	844 (3%)	47 (1%)	
Other continuous medications	1,325 (5%)	166 (3%)	
Any cardiac mechanical support	848 (3%)	44 (1%)	<0.001
Any drains	1,294 (4%)	59 (1%)	<0.001
Central venous or arterial line	5,345 (18%)	777 (12%)	<0.001
Blood transfusion <72 h postcath	1,970 (7%)	203 (3%)	<0.001

Values are n (%).

ETT = endotracheal tube; IV = intravenous; PGE = prostaglandin E; PREDIC^3^T = procedural risk in congenital cardiac catheterization; other abbreviations as in Table 1.

∗ Case durations below the 1st percentile or above the 99th percentile are excluded within each cohort.

Certain higher-risk PREDIC^3^T category 4/5 cases, for example, PDA dilation and/or stenting (4.4% IQIC, 3.3% C3PO) and ventricular septal defect (VSD) device closure (4.1% IQIC, 0.3% C3PO), were performed at a higher rate in IQIC (Supplemental Table 3). Other high-risk cases such as pulmonary vein dilation and/or stent procedures were uncommon in the IQIC cohort (0.2%) compared to the C3PO cohort (4.3%) (Supplemental Table 3).

Overall, the procedure duration was longer in the C3PO cohort. The median case duration for C3PO cases was 1.5 hours, compared to 0.8 hours in IQIC-CHDCR. Notably, 11% of cases in C3PO had a duration of more than 3 hours, in contrast to 1% of IQIC-CHDCR cases (Table 3).

### Procedural resource utilization

Overall, higher-level clinical resources were more commonly utilized in C3PO centers. This includes general anesthesia for sedation (93% C3PO, 72% IQIC) and intubation or/and tracheostomy (92% C3PO, 70% IQIC) in addition to the utilization of IV medications, including vasoactive medications (22% C3PO, 4% IQIC). More than double the number of patients received a blood transfusion in C3PO compared to IQIC (7% and 3%, respectively) (Table 3).

### Hemodynamics and hemodynamic vulnerability score

Of the abnormal hemodynamic values, Qp:Qs > 1.5 was more prevalent in IQIC than in C3PO (19% and 10%, respectively), while others, such as pulmonary vascular resistance >3 indexed wood unit, were noted more frequently in C3PO (14% C3PO, 9% IQIC) (Table 4).

**Table 4:** Hemodynamic Variables and Hemodynamic Vulnerability Score

	C3PO (n = 28,957)		IQIC-CHDCR (n = 6,485)		*P* Value
Abnormal hemodynamic indicator variables^a^ (n = 28,905, n = 6,479)					
Systemic arterial saturation	BiV: 6,269 (31%)	SV: 2,210 (26%)	BiV: 1,262 (23%)	SV: 259 (30%)	<0.001
Mixed venous saturation	BiV: 2,379 (12%)	SV: 1,063 (12%)	BiV: 378 (7%)	SV: 121 (14%)	<0.001
Pulmonary artery pressure	BiV: 3,316 (16%)	SV: 2,050 (24%)	BiV: 714 (13%)	SV: 152 (17%)	<0.001
Systemic ventricle end-diastolic pressure	1,251 (4%)		159 (2%)		<0.001
Qp:Qs	2,955 (10%)		1,194 (19%)		<0.001
Pulmonary vascular resistance	3,974 (14%)		614 (9%)		<0.001
Hemodynamic vulnerability score (n = 28,905, n = 6,330)					
0	14,901 (52%)		3,399 (54%)		<0.001
1	5,323 (18%)		1,561 (25%)		
2	4,576 (16%)		641 (10%)		
≥3	4,105 (14%)		729 (11%)		

Values are n (%). Systemic arterial saturation: BiV: <95%, SV: <78%.

Mixed venous saturation: BiV: <60%, SV: <50%.

Pulmonary artery pressure: BiV: ≥45, SV: ≥17 mm Hg.

Systemic ventricle end diastolic pressure: ≥18 mm Hg.

Qp:Qs: >1.5.

Pulmonary vascular resistance: >3 indexed wood unit.

BiV = biventricular; Qp:Qs = ratio of pulmonary to systemic flow; SV = single ventricle; other abbreviations as in Table 1.

a Thresholds for abnormality.

HVS exhibited significant differences between the 2 cohorts. Lower scores of 0 to 1 were more frequent in IQIC (79% IQIC, 70% C3PO), and higher scores of 2 and ≥3 were less frequent (21% IQIC, 30% C3PO) (Table 4).

### Procedural outcomes

When assessing PREDIC^3^T risk categories, increased frequencies of CMAE were seen in higher categories; however, there were differences among the 2 cohorts. Within low-risk categories 1 and 2, the CMAE rate in C3PO was 2.4% compared to 1.2% in IQIC. In high-risk categories 4/5, the CMAE rate in C3PO was 7.1%, and 2.8% in IQIC (Table 5). Additional details regarding CMAE rates by each case type are detailed in Supplemental Table 4.

**Table 5:** Occurrence of Clinically Meaningful Adverse Events by Age Group and PREDIC^3^T Case Type Risk Category

	C3PO		IQIC-CHDCR		*P* Value
	m	Highest Severity Level 3bc/4/5	m	Highest Severity Level 3bc/4/5	
Age group					
≤30 d	2,955	187 (6.3%)	367	17 (4.6%)	0.25
31 d to <1 y	7,339	344 (4.7%)	1,242	37 (3.0%)	0.006
1 to 18 y	15,203	454 (3.0%)	4,271	37 (0.9%)	<0.001
≥19 y	3,460	135 (3.9%)	605	7 (1.2%)	<0.001
PREDIC^3^T case type risk category					
Category 1	7,217	120 (1.7%)	2,208	15 (0.7%)	<0.001
Category 2	7,252	221 (3.1%)	2,477	40 (1.6%)	<0.001
Category 3	4,987	152 (3.1%)	670	9 (1.3%)	0.009
Category 4	4,383	303 (6.9%)	415	14 (3.4%)	0.004
Category 5	2,652	197 (7.4%)	372	8 (2.2%)	<0.001
Not assigned	2,466	127 (5.2%)	343	12 (3.5%)	0.23

Values are n (%).

PREDIC^3^T = procedural risk in congenital cardiac catheterization; other abbreviations as in Table 1.

Comparison of AE rates between the C3PO and IQIC cohorts reveals notable differences in procedural outcomes (Table 6). The incidence of the CMAE was significantly higher in the C3PO cohort compared to the IQIC cohort (3.9% vs 1.5%; *P* < 0.001). Correspondingly, the number of these AEs per 1,000 patients was also greater in the C3PO cohort (44.2 vs 30.1; *P* < 0.001). AEs of the most severe levels (4/5) differed across the cohorts, with rates of 1.5% in the C3PO cohort and 0.9% in the IQIC cohort (*P* < 0.001); however, the number of these events per 1,000 patients did not differ significantly (17.3 vs 18.5; *P* = 0.51).

**Table 6:** Procedure Outcomes

	C3PO (n = 28,957)	IQIC-CHDCR (n = 6,485)	*P* Value
Adverse events by severity			
Highest severity level 3bc/4/5 (CMAE)	1,120 (3.9%)	98 (1.5%)	<0.001
Number of level 3bc/4/5 AEs	1,279	195	
Number of level 3bc/4/5 AEs per 1,000 patients	44.2	30.1	<0.001
Highest severity level 4/5	432 (1.5%)	58 (0.9%)	<0.001
Number of level 4/5 AEs	502	120	
Number of level 4/5 AEs per 1,000 patients	17.3	18.5	0.51
Mortality			
Total deaths^a^	131 (0.5%)	48 (0.7%)	<0.001
Death within 72 h	108	42	
Death as a result of adverse event	24	7	
Death in lab	6	2	
Failure to rescue^b^			
Percent deaths among cases with CMAE	2.1%	7.1%	0.009

Values are n (%).

AE = adverse event; CMAE = clinically meaningful adverse event; other abbreviations as in Table 1.

a Total deaths refer to death within 72 h, death due to AE, and/or patients who died in the lab. The variable of death within 72 h is independent of whether or not it was the result of an AE.

b Failure to rescue is defined as the number of deaths as a result of AE divided by the number of patients with a level 3bc/4/5 AE.

Mortality rates were slightly lower in the C3PO cohort (0.5% vs 0.7%; *P* < 0.001). Among cases involving CMAE, the percentage of deaths among cases with a CMAE (failure to rescue) was notably lower in the C3PO cohort compared to the IQIC cohort (2.1% vs 7.1%; *P* = 0.009). Upon expert physician review, further scrutiny of the relationship between AEs and the availability of backup support such as ECMO revealed that all 7 deaths attributed to level 5 AEs in the IQIC cohort were potentially associated with lack of life-saving support.

Evaluating the CMAE types, access-related problems were noted to be almost twice as frequent in IQIC (12.0%) compared to C3PO (6.8%). Similarly, device/stent-related problems were more common in IQIC than C3PO (18.0% and 12.6%, respectively). However, events related to the respiratory system were more frequent in C3PO (23.3% compared to 16.0% in IQIC) (Supplemental Table 5).

**Figure 1:**
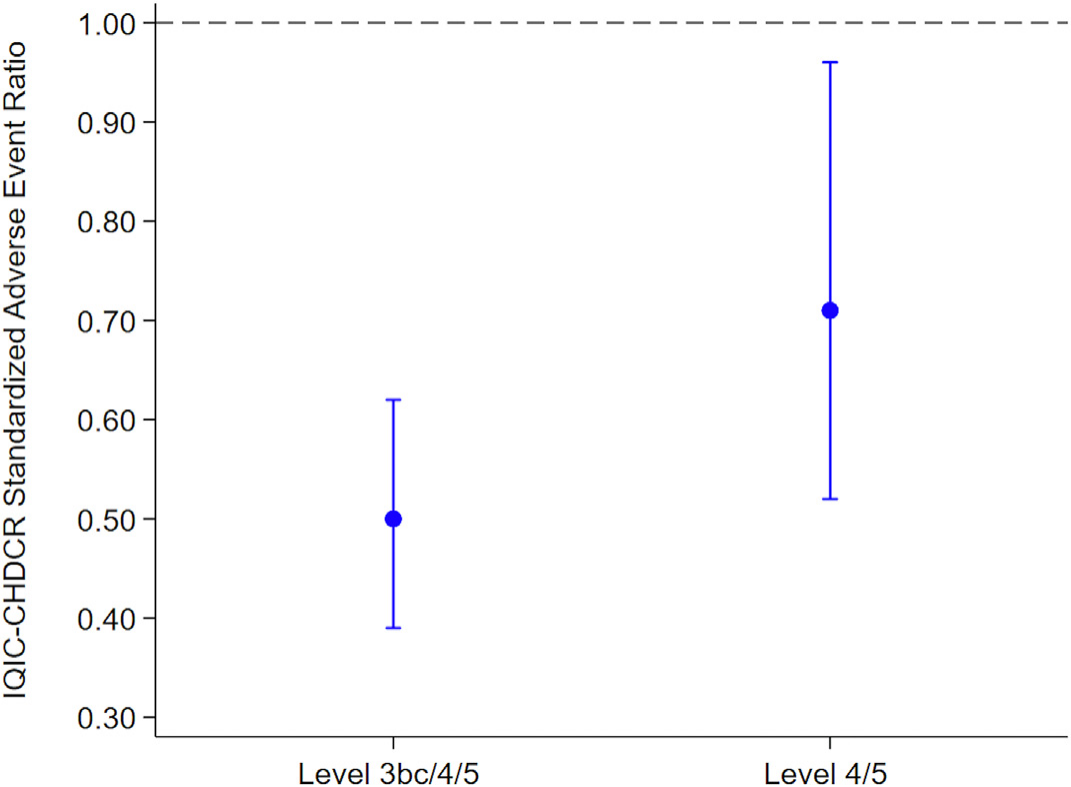
**Standardized Adverse Event Ratios for Clinically Meaningful Adverse Events and Severity Level 4/5 Adverse Events, International Quality Improvement Collaborative Relative to the Congenital Cardiac Catheterization Project on Outcomes** IQIC-CHDCR = International Quality Improvement Collaborative—Congenital Heart Disease Catheterization Registry.

After applying the CHARM-II risk-adjustment model to all eligible cases in both the C3PO and IQIC cohorts, rates of both CMAE and severity level 4/5 events remained lower in the IQIC cohort.^12^ For CMAE, the SAER was 0.50 (95% CI: 0.39-0.62) compared to the C3PO cohort, and for severity level 4/5 events, the SAER was 0.71 (95% CI: 0.52-0.96) (Figure 1).

## Discussion

This retrospective cohort study offers a comprehensive global perspective on CCC programs by comparing data from 2 distinct registries: the C3PO registry, which represents centers in the United States, and the IQIC-CHDCR, exclusively focused on LMICs since its inception in 2019. Our findings reveal that cases in the C3PO registry typically involved higher-risk patient profiles and procedural complexities, as indicated by a greater prevalence of PREDIC^3^T categories 3 to 5 and elevated markers of hemodynamic vulnerability. Additionally, despite the higher incidence of unadjusted CMAEs in the C3PO cohort, the mortality rate was notably lower than that observed in the IQIC cohort, where a higher failure-to-rescue rate creates an opportunity for targeted resource improvements. This analysis highlights disparities in risk profiles and outcomes between high-resource environments and LMIC settings while affirming the safety of CCC in IQIC through its favorable SAER and CMAE incidence rates per 1,000 patients.

The patient characteristics across the 2 cohorts reveal significant differences, notably in the younger age groups, the higher incidence of genetic syndromes, noncardiac comorbidities, and a history of prior surgeries or cardiac catheterizations, with a significant prevalence of SV physiology in the C3PO cohort. These variances likely mirror the advanced resources and well-established health care infrastructures typical of HICs, as opposed to those in LMICs. Factors such as delayed diagnoses, the unavailability of specialized genetic and chromosomal testing, and the lack of adequate infrastructure for timely referrals to specialized centers for complex neonatal surgical management contribute to these disparities. Furthermore, the economic constraints prevalent in LMICs often necessitate that families choose palliative or noninterventional treatments for complex conditions.^1^^,^^5^^,^^14^^,^^15^ Additionally, the predominance of lower-risk, relatively straightforward curative interventions in IQIC sites highlights a cost-effective approach, facilitating broader access to essential care and showcasing a key area where LMICs can achieve significant improvements in patient outcomes through targeted health care strategies.

Significant differences in the characteristics of clinical care were evident between the cohorts, highlighting divergent management practices and health care delivery models driven by the disparate availability of resources, case mix complexity, and institutional capacities. IQIC centers demonstrated a higher frequency of electively scheduled procedures compared to C3PO, reflecting both constraints in resource availability along with deliberate cost-effective strategies. This is further exemplified by the majority of IQIC cases being managed in general wards, contrasting with the more frequent ICU admissions and stepdown unit utilizations in C3PO centers. Additionally, the need for more intensive resources during procedures, such as general anesthesia and airway management, was notably higher in C3PO centers. These differences provide an opportunity for mutual learning between LMICs and HICs, as certain LMIC strategies may offer insights into managing cases safely and effectively with fewer resources. Further work to better understand these disparities can provide valuable insights into how case complexity and resource allocation influence outcomes, offering potential pathways for collaborative learning and quality improvement initiatives that enhance global care standards.

While diagnostic and low-risk procedures (PREDIC^3^T categories 1 and 2) were predominant in the IQIC cohort, a notable proportion of high-risk procedures, such as PDA dilation/stenting (IQIC 4.4% vs C3PO 3.3%), VSD closure (IQIC 4.1% vs C3PO 0.3%), and atretic valve perforation/valvotomy (IQIC 0.2% vs C3PO 0.1%), were also conducted. This trend suggests a reliance on catheter-based treatments in LMICs, potentially due to limited neonatal cardiac surgery resources or family preference for nonsurgical interventions. Conversely, other complex procedures like branch pulmonary artery dilation/stenting, pulmonary vein interventions, and transcatheter pulmonary valve implants were less common in the IQIC cohort, which may reflect a shortage of specialized expertise and the high costs associated with advanced catheterization devices necessary for these therapies.

Procedure durations were notably shorter in LMICs compared to the U.S. registries, a trend observed across various types of procedures and anesthesia methods. Several factors may contribute to this difference. For instance, the sharing of resources with other specialists often imposes time constraints on operators, prompting them to focus on essential aspects of the procedure while minimizing hemodynamic data collection or other less critical elements. Interestingly, these shorter procedure times might inadvertently reduce the risk of AEs and resource utilization, a potential silver lining in resource-constrained settings. A thorough investigation into the effects of procedure duration could illuminate additional factors contributing to these efficiencies and reveal potential compromises made during shorter procedures. Insights gained could benefit cardiac catheterization laboratories worldwide, regardless of their resource levels or geographic locations.

CMAE rates were higher in the C3PO cohort (3.9% vs 1.5% in IQIC), as were severity level 4/5 AE rates (1.5% vs 0.9%), though there was no significant difference in level 4/5 rates per 1,000 patients. Failure-to-rescue was notably higher in the IQIC cohort (7.1% vs 2.1% in C3PO, *P* = 0.009). These differences likely reflect greater patient and procedural complexity in C3PO, supported by a robust safety net that allows for higher-risk procedures. In contrast, IQIC-CHDCR, as a newer registry, demonstrated lower rates of AEs, particularly low-severity level 3 events, which may be partly due to limited follow-up and resource constraints where limited follow-up hinders the identification of postprocedure complications. Higher CMAE rates for isolated procedures such as PDA and VSD device closures in C3PO may suggest a more aggressive approach to achieving technical success. These variations are likely multifactorial, influenced by case complexity and reporting rigor. Ongoing data collection and the development of new metrics for patient safety and technical success will enhance understanding and refine the analysis of procedural performance in both cohorts.^12^^,^^16^^,^^17^ In this study, mortality was defined as any death occurring within 72 hours postcatheterization. Although the AE profiles were similar, the mortality rate was notably higher in the IQIC registry compared to the C3PO cohort. More than two-thirds of these cases lacked sufficient data to determine the relationship between the AE and the cause of mortality, highlighting a critical need for enhanced data capture and analysis. Better understanding the causes of mortality and their potential links to complications is crucial for providing valuable insights into the disparities in mortality rates between the cohorts.

The concept of “failure to rescue”—the inability to prevent death or a higher-severity AE after the occurrence of a complication—is particularly relevant in the IQIC context. All deaths attributed to AEs within IQIC were considered preventable upon expert review if adequate resources such as ECMO support were available. This shortage of essential resources, including specialized catheterization equipment, backup surgical options, and immediate access to an operating room, coupled with the lower case volumes, likely contributes to the higher mortality rate observed in the IQIC cohort. Understanding these dynamics can inform targeted improvements in patient care and resource allocation, potentially reducing mortality rates in LMIC settings.

This report highlights the significant challenges faced by LMIC centers, particularly those stemming from resource limitations. However, categorizing programs solely based on geographic location as low- or high-resourced risks misrepresents. Such a simplistic classification fails to acknowledge the advanced capabilities of some programs in low- to middle-income countries and the handling of less complex CHD cases by labs in high-income countries, potentially skewing performance comparisons. A more sophisticated approach would categorize cardiac catheterization labs based on nuanced factors such as patient demographics, disease spectrum, health system sophistication, and specific resources. Additionally, the disparities in case mix complexity and resource availability necessitate the development of tailored risk adjustment methodologies for LMICs. Implementing these measures will enhance our understanding of CHD management and foster more effective interventions and outcomes in these settings.

### Study Limitations

Currently, the generalizability of findings from this study is limited due to a low participation rate in the IQIC centers, where 19 out of 74 (22%) enrolled sites in the IQIC surgical database are entering data in IQIC-CHDCR. Similarly, HIC sites are limited to the U.S. centers enrolled in C3PO and may not reflect practices in other HICs with differing health care infrastructures and resources. Additionally, LMIC sites that voluntarily submit data and engage in quality improvement initiatives, such as those in our study, are likely more motivated toward ensuring patient safety and represent the highest-performing institutions. Therefore, these sites may not accurately represent the average LMIC site, potentially skewing perceptions of broader regional practices. Encouraging broader participation, particularly from LMICs, and ensuring complete data entry are crucial steps that could significantly enhance the generalizability and applicability of future studies.

It is noteworthy that C3PO, as a collaborative effort, has matured over time, demonstrating its ability to capture standardized and maximum data and improve outcomes through quality improvement initiatives. In contrast, IQIC-CHDCR is a relatively new registry, and its capacity to consistently capture standardized and maximally available outcome data is still in the maturing phase. This is evident in the observed differences in rates of AE reporting and, notably, a significant portion (approximately 33%) of radiation data that were not available for analysis.

The impact of the COVID-19 pandemic on procedural volumes and case mix varied significantly across centers and time periods, particularly between international and U.S. sites.^18^^,^^19^ While 2020 data were included to provide a comprehensive dataset, the variability in pandemic-related effects may introduce heterogeneity that may influence findings during this timeframe.

Being a retrospective review of variables entered in the registries, a deeper dive into causes and attributability of AEs and mortality was not possible. Similarly, the failure-to-rescue analysis in this study is limited by the absence of a specific failure-to-rescue variable. This approach assumes, rather than confirms, a direct progression from lower-severity events to catastrophic outcomes. Future data collection should incorporate variables that explicitly link lower-severity events to subsequent higher-severity outcomes to improve the accuracy and interpretability of failure-to-rescue assessments. Furthermore, application of the CHARM-II methodology to the IQIC cohort for SAER analysis needs to be further studied, as this methodology may not fully account for key variables that are uniquely relevant in LMIC settings.

## Conclusions

Leveraging the harmonization of databases facilitated a valuable and insightful comparison between C3PO and IQIC-CHDCR. The analysis revealed a younger and more clinically complex patient population in C3PO compared to IQIC. Despite these differences, procedures performed in the IQIC cohort demonstrated safety, as evidenced by a favorable SAER and lower CMAE rates. However, the higher failure-to-rescue rate in IQIC suggests an opportunity to further enhance care and resource availability. These findings highlight the essential role of continuous research and international collaboration in improving patient outcomes. Future efforts to expand participation in LMICs and to include HIC centers outside of the United States are necessary to improve the generalizability of these findings, providing more inclusive global insights. By deepening our understanding of these differences, this study paves the way for targeted interventions designed to enhance care quality and patient safety across diverse health care settings.

## Funding support and author disclosures

The authors have reported that they have no relationships relevant to the contents of this paper to disclose.
