# Supplementary material for: Comparative Congenital Cardiac Catheterization Registry Analysis From the United States and Low- and Middle-Income Countries
Source: JACC Adv. 2025 Mar 20;4(4):101649. doi: 10.1016/j.jacadv.2025.101649 (PMC11976248; doi:10.1016/j.jacadv.2025.101649)
Supplement: Supplemental data [file mmc1.pdf]

Supplemental Appendix

Supplemental Table 1: Participating Sites by Year

**IQIC Sites**	**2019**	**2020**	**2021**	**2022**
Aga Khan University Hospital	●	●	●	●
Clinica Cardio VID	●			
Fundacion Cardioinfantil de Bogota	●			
Hospital Serdang		●	●	
Institut Jantung Negara		●	●	
Institute of General & Urgent Surgery	●			
Instituto do Coracao (InCor)	●			
Instituto Nacional Ignacio Chávez	●			
Jakaya Kikwete Cardiac Institute			●	●
Mother and Child Health Institute			●	
Narayana Superspeciality Hospital		●	●	●
National Heart Hospital	●	●	●	●
Nhi Dong 1		●		●
Rabindranath Tagore International Institute of Cardiac Sciences (RTIICS)			●	●
Red Cross War Memorial	●	●		●
SRCC Children's Hospital				●
TAIYARI AC	●			
TEDA International		●		
Uganda Heart Institute	●			
**C3PO Sites**	**2019**	**2020**	**2021**	**2022**
Arnold Palmer Children’s Hospital – Orlando Health		●	●	●
Boston Children’s Hospital	●	●	●	●
C.S. Mott Children’s Hospital – Michigan Medicine	●	●	●	●
Children’s Health			●	●
Children’s Hospital of Philadelphia	●	●	●	●
Children’s Wisconsin	●	●	●	●
Cincinnati Children’s Hospital Medical Center	●	●	●	●
Cleveland Clinic Children’s Hospital				●
Le Bonheur Children’s Hospital		●	●	●
Monroe Carell Jr. Children’s Hospital at Vanderbilt	●	●	●	●
Nationwide Children’s Hospital	●	●	●	●
Norton Children’s Hospital			●	●
NYP Morgan Stanley Children’s Hospital	●	●	●	●
Rady Children’s Hospital	●	●	●	●
Rainbow Babies’ and Children’s Hospital		●	●	●
St Louis Children’s Hospital	●	●	●	●
University of Virginia Health Children’s Hospital	●	●	●	●

UPMC Children's Hospital of Pittsburgh	●	●	●	●
Weill Cornell Medicine Children's Hospital	●	●	●	●

Supplemental Table 2: Key Variables Audited

Variable	C3PO	IQIC-CHDCR
**Patient/Procedure Characteristics**	Date of catheterization<br>Operator<br>Age at time of procedure<br>Weight at time of procedure<br>Case type performed.<br>Total DAP<br>Total cath time	Date of catheterization<br>Age at time of procedure<br>Case type performed.<br>Total DAP
**Haemodynamics**	Single or bi-ventricle physiology<br>Systemic arterial saturation<br>Mixed venous saturation<br>Systemic ventricle end diastolic pressure.<br>Pulmonary artery pressure<br>Qp:Qs<br>Pulmonary vascular resistance	Single or bi-ventricle physiology<br>Systemic arterial saturation<br>Mixed venous saturation<br>Systemic ventricle end diastolic pressure<br>Pulmonary artery pressure<br>Qp: Qs<br>Pulmonary vascular resistance
**Clinical Characteristics**	Discharge > 48 hours post-cath	Discharge > 48 hours post-cath.<br>Alive at 72 hours post-cath
**Adverse Events**	Occurrence, type, and severity	Occurrence, type, and severity
**Procedural Resources**	Airway management<br>Ventilation<br>Sedation<br>Lines/drains<br>Mechanical support<br>IV medications<br>Nursing assignment	Airway management<br>Sedation

Supplemental Table 3: PREDIC³T Case Type Risk Categories

**PREDIC³T Case Type**	**C3PO** (N = 26491) *N (%)*	**IQIC-CHDCR** (N = 6142) *N (%)*
**Category 1**		
Fontan fenestration/baffle leak device closure	71 (0.3%)	7 (0.1%)
Pulmonary valvotomy, age >30 days	592 (2.2%)	269 (4.4%)
Diagnostic only, age ≥1 year	6554 (24.7%)	1932 (31.5%)
**Category 2**		
ASD or PFO device closure	1384 (5.2%)	753 (12.3%)
Venous collateral device or coil occlusion	630 (2.4%)	26 (0.4%)
PDA device or coil closure	2787 (10.5%)	1277 (20.8%)
Diagnostic only, age >30 days to <1 year	2399 (9.1%)	394 (6.4%)
Pulmonary valvotomy + procedure, age >30 days	52 (0.2%)	27 (0.4%)
**Category 3**		
Pulmonary artery (only 1 vessel)	1240 (4.7%)	136 (2.2%)
Fontan fenestration/baffle leak device closure + procedure	57 (1.4%)	1 (<0.1%)
Aorta (coarctation) dilation and/or stent	846 (0.6%)	171 (2.8%)
Systemic pulmonary collateral device or coil closure	1235 (2.0%)	171 (2.8%)
Pulmonary valvotomy, age ≤30 days	358 (2.2%)	36 (0.6%)
Pulmonary artery (only 1 vessel) + RVOT conduit dilation/stent	146 (0.6%)	1 (<0.1%)
Atrial septostomy	517 (2.0%)	72 (1.2%)
Diagnostic only, age ≤30 days	588 (2.4%)	82 (1.3%)
**Category 4**		
Pulmonary artery (only 1 vessel) + procedure	544 (2.1%)	9 (0.2%)
ASD or PFO device closure + procedure	86 (0.3%)	21 (0.3%)
Pulmonary vein dilation and/or stent	1135 (4.3%)	10 (0.2%)
Pulmonary artery (≥2 vessels)	1037 (3.9%)	39 (0.6%)
RVOT conduit dilation and/or stent	695 (2.6%)	69 (1.1%)
PDA dilation and/or stent	886 (3.3%)	267 (4.4%)
**Category 5**		
Aorta (coarctation) dilation and/or stent + procedure	191 (0.7%)	4 (0.1%)
Aortic valvotomy, age >30 days	351 (1.3%)	57 (0.9%)
Pulmonary artery (≥2 vessels) + RVOT and/or other procedure	338 (1.3%)	2 (<0.1%)
Aortic valvotomy, age ≤30 days	112 (0.4%)	23 (0.4%)
VSD device closure	71 (0.3%)	254 (4.1%)
Mitral valvotomy	33 (0.1%)	5 (0.1%)

Atrial septostomy + procedure	122 (0.5%)	2 (<0.1%)
TPV implantation	1236 (4.7%)	10 (0.2%)
Atrial septum static dilation and/or stent placement	166 (0.6%)	3 (0.1%)
Atretic valve perforation and/or valvotomy	32 (0.1%)	12 (0.2%)

Supplemental Table 4: Adverse Events by PREDIC^{3}T Case Type

	Number of Cases		Highest Severity Level 3bc/4/5	
**PREDIC^{3}T Case Type**	**C3PO**	**IQIC-CHDCR**	**C3PO**	**IQIC-CHDCR**
	N		*N (%)*	
Category 1	7217	2208	120 (1.7%)	15 (0.7%)
Fontan fenestration/baffle leak device closure	71	7	0 (0.0%)	0 (0.0%)
Pulmonary valvotomy, age >30 days	592	269	11 (1.9%)	3 (1.1%)
Diagnostic only, age ≥1 year	6554	1932	109 (1.7%)	12 (0.6%)
Category 2	7252	2477	221 (3.1%)	40 (1.6%)
ASD or PFO device closure	1384	753	28 (2.0%)	9 (1.2%)
Venous collateral device or coil occlusion	630	26	19 (3.0%)	0 (0.0%)
PDA device or coil closure	2787	1277	88 (3.2%)	18 (1.4%)
Diagnostic only, age >30 days to <1 year	2399	394	83 (3.5%)	13 (3.3%)
Pulmonary valvotomy + procedure, age >30 days	52	27	3 (5.8%)	0 (0.0%)
Category 3	4987	670	152 (3.1%)	9 (1.3%)
Pulmonary artery (only 1 vessel)	1240	136	39 (3.2%)	2 (1.5%)
Fontan fenestration/baffle leak device closure + procedure	57	1	1 (1.8%)	0 (0.0%)
Aorta (coarctation) dilation and/or stent	846	171	25 (3.0%)	4 (2.3%)
Systemic pulmonary collateral device or coil closure	1235	171	33 (2.7%)	0 (0.0%)
Pulmonary valvotomy, age ≤30 days	358	36	12 (3.4%)	0 (0.0%)
Pulmonary artery (only 1 vessel) + RVOT conduit dilation/stent	146	1	8 (5.5%)	0 (0.0%)
Atrial septostomy	517	72	15 (2.9%)	1 (1.4%)
Diagnostic only, age ≤30 days	588	82	19 (3.2%)	2 (2.4%)
Category 4	4383	415	303 (6.9%)	14 (3.4%)
Pulmonary artery (only 1 vessel) + procedure	544	9	17 (3.1%)	0 (0.0%)
ASD or PFO device closure + procedure	86	21	4 (4.7%)	0 (0.0%)
Pulmonary vein dilation and/or stent	1135	10	79 (7.0%)	0 (0.0%)
Pulmonary artery (≥2 vessels)	1037	39	75 (7.2%)	1 (2.6%)
RVOT conduit dilation and/or stent	695	69	50 (7.2%)	2 (2.9%)
PDA dilation and/or stent	886	267	78 (8.8%)	11 (4.1%)
Category 5	2652	372	197 (7.4%)	8 (2.2%)
Aorta (coarctation) dilation and/or stent + procedure	191	4	9 (4.7%)	1 (25.0%)
Aortic valvotomy, age >30 days	351	57	17 (4.8%)	1 (1.8%)
Pulmonary artery (≥2 vessels) + RVOT and/or other procedure	338	2	33 (9.8%)	1 (50.0%)

Aortic valvotomy, age ≤30 days	112	23	13 (11.6%)	2 (8.7%)
VSD device closure	71	254	11 (15.5%)	1 (0.4%)
Mitral valvotomy	33	5	1 (3.0%)	0 (0.0%)
Atrial septostomy + procedure	122	2	15 (12.3%)	0 (0.0%)
TPV implantation	1236	10	85 (6.9%)	0 (0.0%)
Atrial septum static dilation and/or stent placement	166	3	8 (4.8%)	0 (0.0%)
Atretic valve perforation and/or valvotomy	32	12	5 (15.6%)	2 (16.7%)

Supplemental Table 5: Adverse Events by System

**AE System (CMAE)**	**C3PO** n = 1143	**IQIC-CHDCR** n = 100	*P-Value*
Access Problem	78 (6.8%)	12 (12.0%)	*<0.001*
Arrhythmia	202 (17.7%)	14 (14.0%)	
Device or Stent Embolization/Malposition	144 (12.6%)	18 (18.0%)	
Hemodynamic Problem (including metabolic acidosis)	57 (5.0%)	9 (9.0%)	
Respiratory System	266 (23.3%)	16 (16.0%)	
Other	396 (34.6%)	31 (31.0%)	

** Note: Cases can have more than AE with events attributed to different systems.*

12
